# Gas injection and brine discharge in rock salt gas storage studied via numerical simulation

**DOI:** 10.1371/journal.pone.0207058

**Published:** 2018-11-28

**Authors:** Jianjun Liu, Yingjie Wang, Kai Xie, Yichen Liu

**Affiliations:** 1 School of Geoscience and Technology, Southwest Petroleum University, Chengdu, China; 2 State Key Laboratory of Geomechanics and Geotechnical Engineering, Institute of Rock & Soil Mechanics, Chinese Academy of Sciences, Wuhan, China; 3 School of Civil Engineering and Architecture, Southwest Petroleum University, Chengdu, China; University of New South Wales, AUSTRALIA

## Abstract

Underground gas storage in rock salt is of great importance for peak-shaving and emergency gas supply. This paper addressed an actual rock salt underground gas storage facility in Jiangsu province, China, as the research project and carried out the following research centered on a detailed geological model, a salt cavern model and the process of gas injection and brine discharge. First, based on the theory of gas-liquid two-phase flow, the authors established a relationship between brine flow and natural gas bubbles under high pressure in the process of brine discharge. Second, the effect of pipe depth on the gas injection and brine discharge was simulated. The objective was mainly to choose the best combination of pipe depth and rate of brine discharge flow based on analysis of the relationship between the brine discharge pipe depth and the flow rate of the residual brine, and the optimal rate was given according to different distances. Third, the effect of residual brine on the gas injection and brine discharge was analyzed. The relationship curves between the maximum velocity on the surface of brine and the distance from the lower end of the brine discharge pipe to the bottom of the gas storage were obtained, and reasonable rates were suggested under different actual working conditions.

## Introduction

There are many advantages to salt cavern underground gas storage in rock salt: for example, the creep of rock salt formations is good, the permeability of rock salt formations is low, the structure of rock salt formations is complete, the hydrogeological conditions are relatively simple, and the caprock is well separated. Rock salt is readily soluble in water, which can reduce construction costs. Therefore, salt cavern gas storage is performed in water-soluble rock salt deposits and has become the most widely used type of natural gas reserve in the world [1–7].

**Fig 1:**
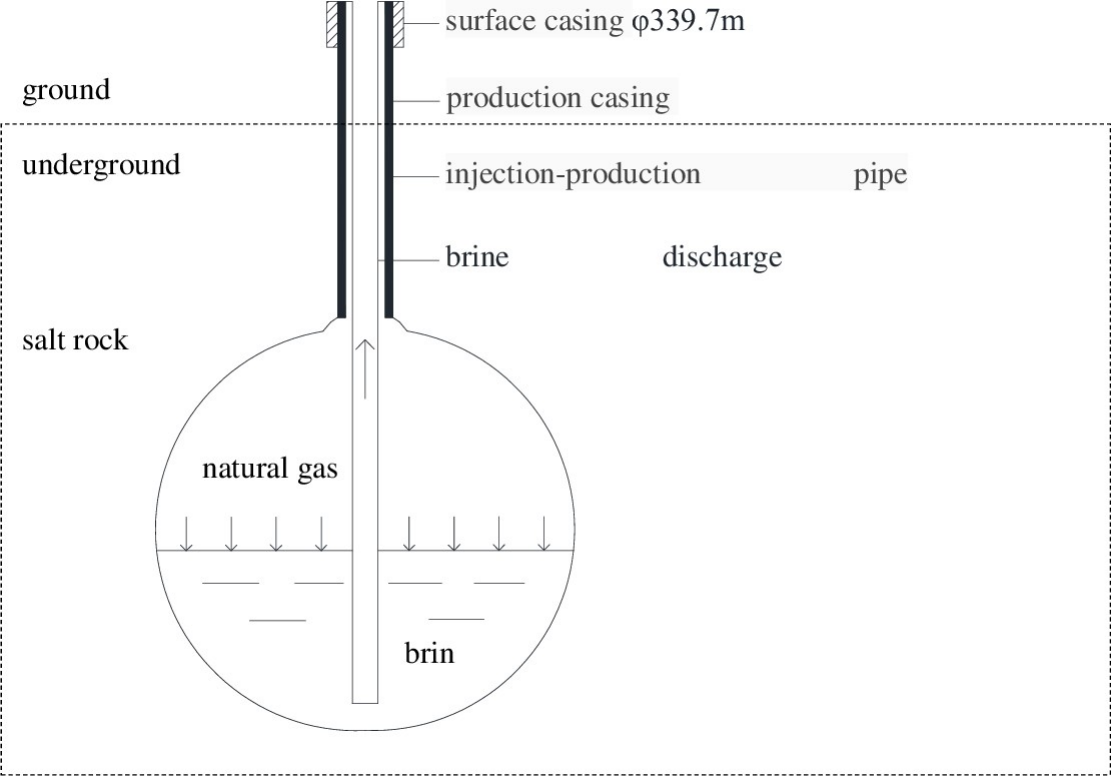
A schematic diagram of gas injection and brine discharge.

The construction of salt cavern underground gas storage includes not only the construction of the supporting ground facilities and pipelines but also drilling and completion, cavity construction, gas injection and brine discharge. At first, according to the design of drilling, the casing, intermediate pipe and central pipe are drilled, and the cavity is built after the installation of the wellhead cavity facility is completed. The rock salt is continuously dissolved as freshwater is pumped into the rock salt layer through the pipes, and the salt cavern is gradually expanded under this artificial control. The process of gas injection and brine discharge are continued after the sealing ability and stability of salt cavern have been evaluated [8–12]. After the formation of the underground salt cavern, natural gas is injected into the cavern to bring out the water, which is the first injection of natural gas into the salt cavern gas storage volume, marking the switch from construction of the production system to operation. Fig 1 is a schematic diagram of the gas injection and brine discharge. The underground natural gas storage is located about 1000 meters underground. The brine is discharged out of the storage after the salt rock is dissolved by clear water injected through the pipeline, and then a cavity with a certain volume and shape is formed underground. Therefore, it is of great significance for the construction of natural gas storage by controlling the position of pipe string and the rate of brine velocity.

## 1 Research background of gas storage

Based on the economic and strategic importance of the underground rock salt reserves, in recent years, research on salt caverns has become more mature in many countries [13–17]. At home and abroad, this field has made great progress, mainly in the numerical simulation and physical test models of the characteristics of the rock salt and the structural stability and long-term stability of rock salt. In the first rock salt discussion meeting, R.W Jessen [18] proposed to measure the shape of the salt cavern at the stage of gas injection and brine discharge and obtain the shape of the salt cavern through the interpretation of seismic data. In 1986, Reda [19] developed a cylindrical cavern through an experiment to simulate the cavity process, which was used to simulate the process of cavity formation by injecting water into the cavity. Based on the mass energy equation of natural gas, Hagorrt [20] established a mathematical model in 1993 to predict intracavity pressure and temperature changes.

Ding [21,22] established a model using heat exchange mass balance and the energy balance equation in the case of a water-soluble cavity and gas injection, which had a great influence on the subsequent injection. The study shows that the worst working conditions for rock salt underground gas storage involve the process of gas injection and brine discharge. Chen [23] combined the actual situation of rock salt gas storage in China with a two-dimensional finite element model that was established to simulate the flow field in the bottom of the nozzle and obtained the safe and reasonable rate of brine removal based on the analysis of the relationship between the maximum velocity of the brine interface and the residual brine in the later stage of gas injection. Cao [24] established a dynamic simulation model for the gas injection and discharge stage of salt cavern gas storage. Yang [25] performed a dynamic analysis on the process of gas injection and injection production for salt cavern gas storage.

In the process of gas injection and brine discharge, the cavity brine and injected natural gas coexist under the high-pressure condition in the rock salt cavern; the main parameters of the research are the gas injection pressure, the pressure of the brine discharge row, the halogen flow rate, and the sonar measurement data of cavity shape. The influence of the depth of the brine injection pipe and the gas injection rate on the removal efficiency is studied mainly by focusing on a two-dimensional finite element model to simulate the results of brine discharge and the effluent halogen effect [26–28], but the actual cavity is much more complicated than the established two-dimensional model. If it is not reasonable to adjust the depth of the exhaust pipe and the gas injection rate, this adjustment would cause the natural gas to be discharged to the surface with the brine, effectively use of the natural gas in the process of gas injection and brine discharge and cause potential harm to the ground personnel and facilities [29–32]. Therefore, it is of great practical significance to study the optimization of gas injection and brine removal to prevent the leakage of natural gas, adjust the construction reasonably, and ensure the safety of the personnel on the ground.

## 2 Fluid dynamics theory in the process of gas injection

In the process of gas injection, the high-pressure brine injected into the pipe is a type of compressible unsteady research object, and the brine in the cavern is a type of incompetent stationary research object. Therefore, the fluid dynamics equations in the process of gas injection can be established according to the laws of mass, momentum and energy conservation of fluid mechanics.

In the process of gas injection, the gas movement in the cavern is an active situation because the natural gas is continuously injected into the gas storage volume. It is assumed that the chamber cavity brine is a steady incompressible fluid so that the continuity equation is as follows:

$$\frac{\partial (\rho u)}{\partial x}+\frac{\partial (\rho v)}{\partial y}+\frac{\partial (\rho w)}{\partial z}=S$$ (1)

where *ρ* is the density of brine; *u*, *v*, and *w* are the components of velocity along the *x-*, *y-*, and *z-*axes; and *S* is the source term.

Assuming that the fluid is a Newtonian fluid, according to the equation for momentum conservation, the equations of motion of the fluid in the stage of gas injection can be obtained:

$$\frac{\partial (\rho u)}{\partial t}+\frac{\partial (\rho uu)}{\partial x}+\frac{\partial (\rho uv)}{\partial y}+\frac{\partial (\rho uw)}{\partial z}=\frac{\partial }{\partial x}(\mu \frac{\partial u}{\partial x})+\frac{\partial }{\partial y}(\mu \frac{\partial u}{\partial y})+\frac{\partial }{\partial z}(\mu \frac{\partial u}{\partial z})-\frac{\partial p}{\partial x}$$ (2)

$$\frac{\partial (\rho v)}{\partial t}+\frac{\partial (\rho vu)}{\partial x}+\frac{\partial (\rho uv)}{\partial y}+\frac{\partial (\rho vw)}{\partial z}=\frac{\partial }{\partial x}(\mu \frac{\partial v}{\partial x})+\frac{\partial }{\partial y}(\mu \frac{\partial v}{\partial y})+\frac{\partial }{\partial z}(\mu \frac{\partial v}{\partial z})-\frac{\partial p}{\partial y}$$ (3)

$$\frac{\partial (\rho w)}{\partial t}+\frac{\partial (\rho wu)}{\partial x}+\frac{\partial (\rho wv)}{\partial y}+\frac{\partial (\rho ww)}{\partial z}=\frac{\partial }{\partial x}(\mu \frac{\partial w}{\partial x})+\frac{\partial }{\partial y}(\mu \frac{\partial w}{\partial y})+\frac{\partial }{\partial z}(\mu \frac{\partial w}{\partial z})-\frac{\partial p}{\partial z}+Fz$$ (4)

where *p* is the pressure on the object to be controlled, *F*_*z*_ is the body force on the *z*-axis, and *μ* is the dynamic viscosity of the fluid.

The equation for the adiabatic steady flow during the process of gas injection can be expressed as follows:

$$gdz+d\left(\frac{p}{\rho }\right)+d\left(\frac{V^{2}}{2}\right)=0$$ (5)

where *V* is the fluid rate in the object to be controlled.

Therefore, the *k-ε* equation is chosen as the turbulence model considering the accuracy and convergence of calculation in the process of gas injection and brine discharge.

## 3 Numerical simulation of gas injection and brine discharge

The comprehensive treatment of the well depth ranges from 12.45–1175.0 m, which consists of the strata of several formations: the Dong Tai Formation, upper San Duo Formation, middle San Duo Formation, lower San Duo Formation, upper Dai Nan Formation, lower Dai Nan Formation, and Fu Ning Formation, as shown in Table 1.

**Table 1:** Table of the geological strata.

Stratum				Depth (m)	Thickness (m)
System	Series	Formation	Symbol		
Quaternary	Pleistocene	Dong Tai	Qd	35.0	35.0
Paleogene	Oligocene to Eocene	San Duo	Es_3_	415.5	380.5
			Es_2_	589.0	173.5
			Es_1_	634.5	45.5
		Dai Nan	Ed_2_	792.0	157.5
			Ed_1_	934.0	142.0
	Eocene~Paleocene	Fu Ning	Ef_4_	1175.0 (not reached)	241.0

**Fig 2:**
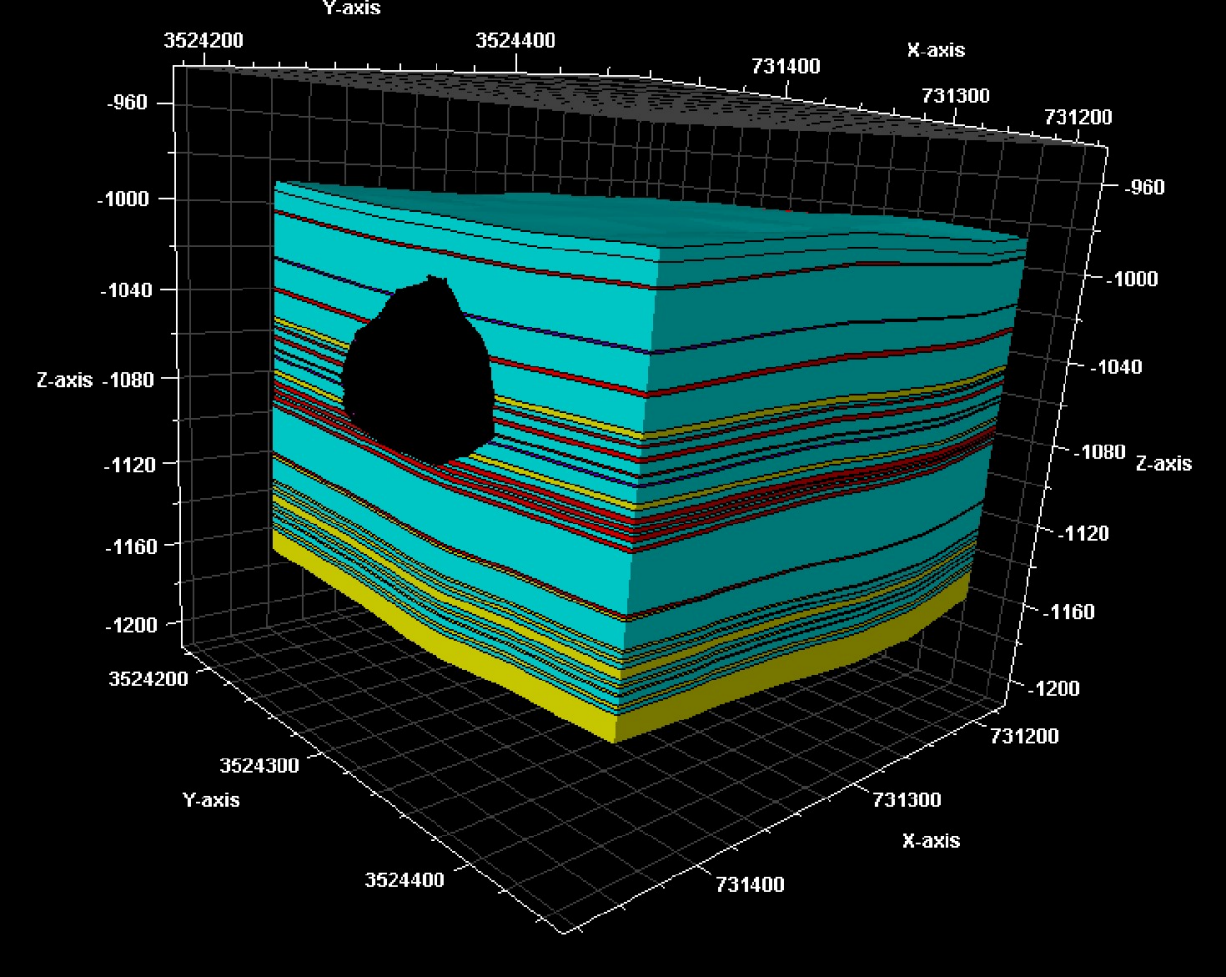
The geological model and the distribution of the rock salt formations.

The depth of the cavity was measured by sonar logging. The depth of the cavity is 1016–1089 meters underground. The geological model of the rock salt gas storage is established by using numerical simulation software. The model is shown in Fig 2.

## 4 Effect of pipe depth on the gas injection and brine discharge

The purpose of the research on the effect of gas injection and brine discharge is mainly to select the best combination of pipe depth and rate of brine discharge flow, based on the analysis of the relationship between the brine discharge pipe depth and the flow rate of the residual brine.

**Fig 3:**
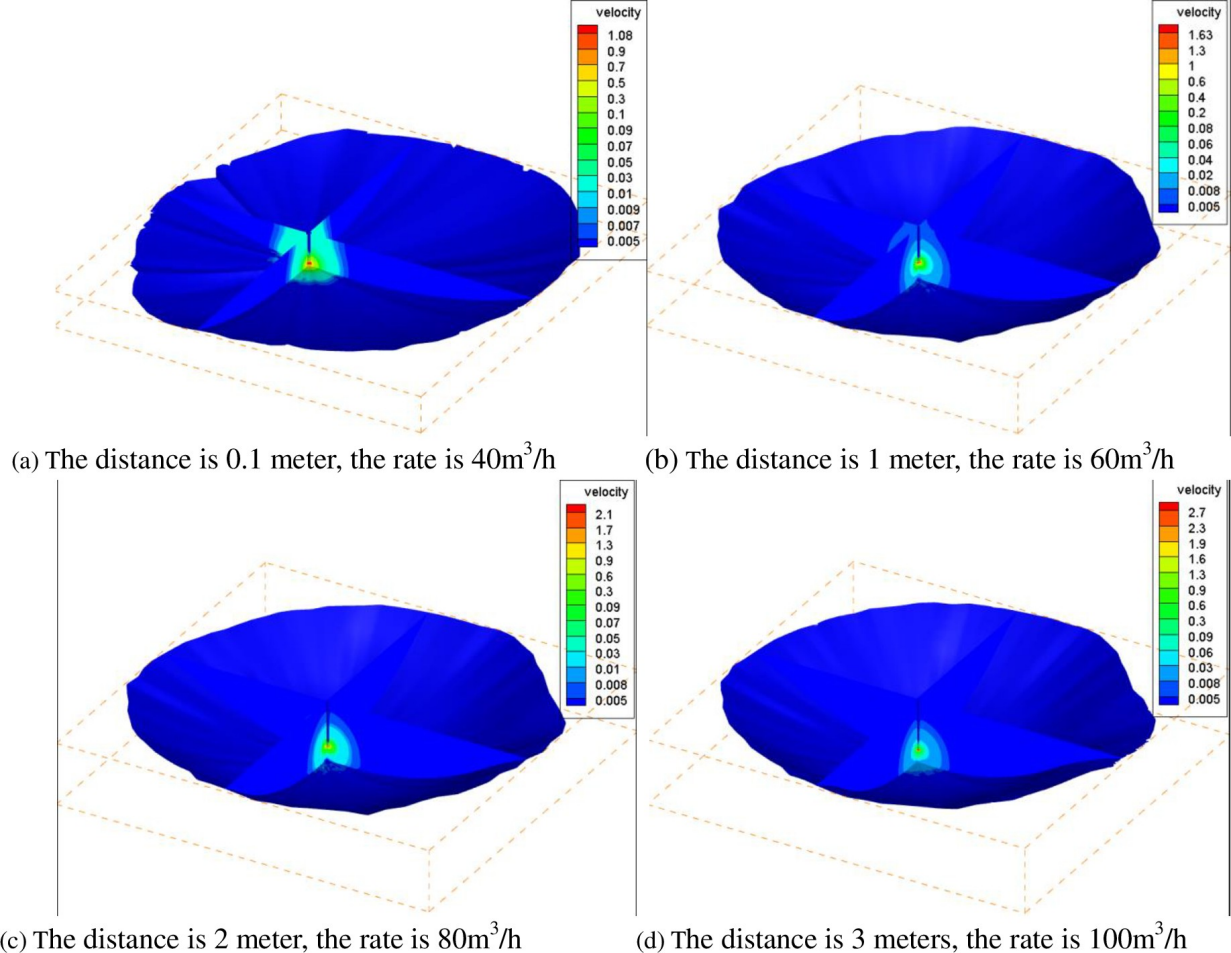
The velocity distributions for different distances from the lower end of the brine discharge pipe to the bottom of gas storage volume and different flow rates of the brine discharge. (a) The distance is 0.1 meter, and the rate is 40 m^3^/h. (b) The distance is 1 meter, and the rate is 60 m^3^/h. (c) The distance is 2 meters, and the rate is 80 m^3^/h. (d) The distance is 3 meters, and the rate is 100 m^3^/h.

In this paper, the models considered the different distances of 0.1 meter, 1 meters, 2 meters and 3 meters from the lower end of the brine discharge pipe to the bottom of the gas storage; the surface of the residual brine is 2 meters higher than the bottom of the brine discharge pipe. Based on the boundary conditions in Fig 1, the distribution of the brine surface velocity and the maximum velocity of the brine under different conditions can be obtained. The results are shown in Table 2 and Fig 3.

**Table 2:** The maximum velocity on the surface of brine (m/s).

rate of flow distance	100 m^3^/h	80 m^3^/h	60 m^3^/h	40 m^3^/h
0.1	0.0225	0.0178	0.0138	0.0090
1	0.0099	0.0079	0.0059	0.0039
2	0.0054	0.0040	0.0028	0.0016
3	0.0036	0.0028	0.0022	0.0014

As shown in Table 2 and Fig 3, in the saturated brine, a natural gas bubble moves upward with a velocity of *V*, and the saturated brine of the gas-liquid interface moves at a velocity of *Vw*; the downward velocity component is *Vwy*. Because the velocity of the flow field gradually decreases from the lower end of the brine discharge pipe to the gas-liquid interface, only when *V*<*Vwy* can the bubbles be transported by the high-speed flow of brine.

**Fig 4:**
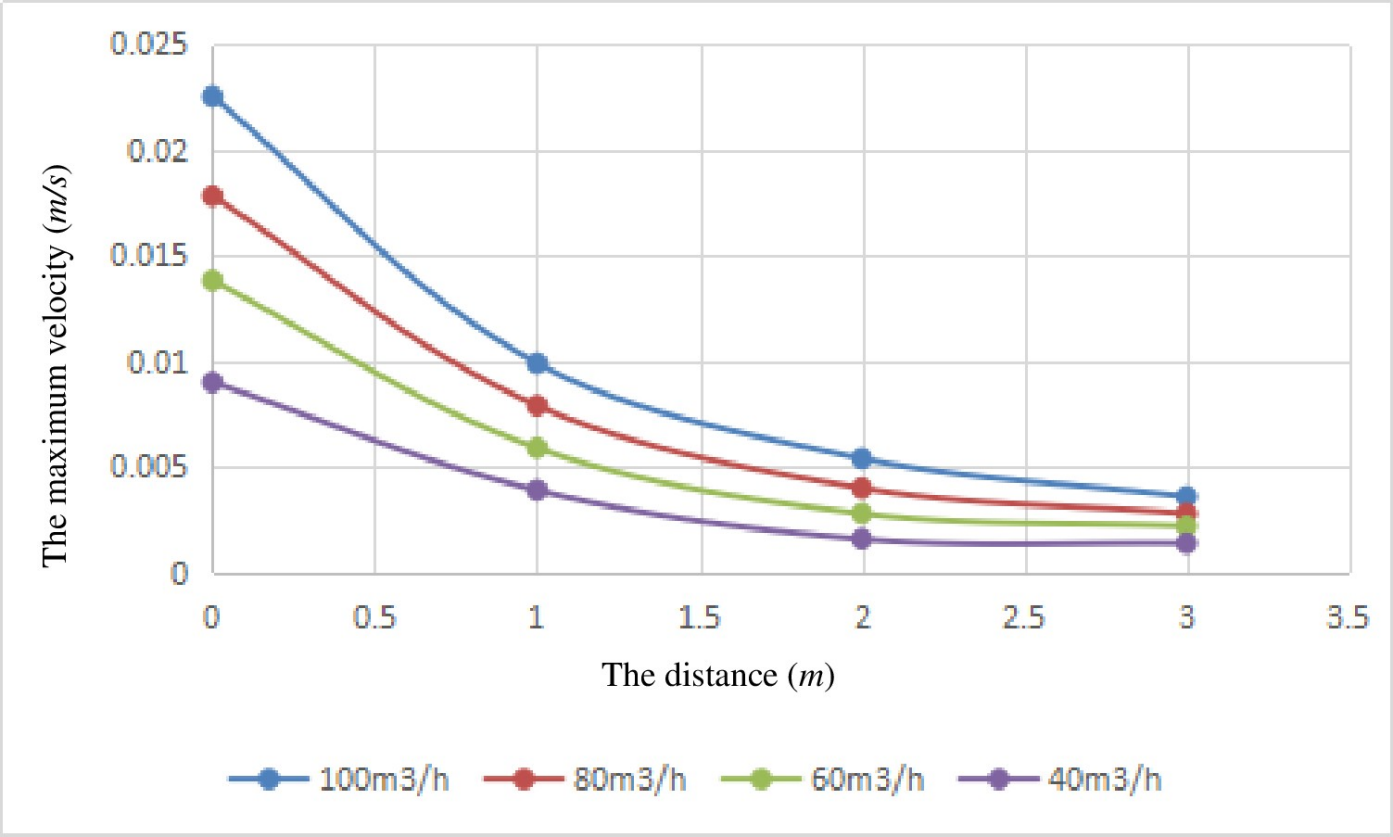
The relationship curves between the velocity of the surface of brine and the distance from the lower end of the brine discharge pipe to the bottom of the gas storage volume.

As seen in Fig 4, these four kinds of operating velocity curve are similar in their variation trend. Under different working conditions, the maximum velocity of the gas-liquid interface increases with the decrease in the distance from the lower end of brine discharge pipe to the bottom of the gas storage volume, and when the distance drops to 1 meter, the change rate of the maximum velocity of the gas-liquid interface increases greatly. The rate of change in velocity of the gas-liquid interface increases gradually with the decrease in the brine flow rate, and it reaches its largest value when the flow rate is 100 m^3^/h. When the distance is held constant, the maximum velocity increases linearly with the increase in the flow rate, and the gradient of the water velocity increases near the brine discharge pipeline.

When the distance between the lower end of the brine discharge pipe and the surface of the brine is 2 meters, based on the basic law of brine-carrying gas bubbles, taking a bubble with a diameter of 0.1 mm as an example, the following conclusions can be drawn: (1) If the distance between the lower end of the brine discharge pipe and the bottom of the gas storage volume is 3 meters, the natural gas bubbles cannot be transported out of the cavern when the flow rate of brine discharge is less than 100 m^3^/h. (2) If the distance between the lower end of the brine discharge pipe and the bottom of the gas storage volume is 2 meters, the natural gas bubbles cannot be transported out of the cavern when the flow rate of brine discharge is less than 80 m^3^/h. (3) If the distance between the lower end of the brine discharge pipe and the bottom of the gas storage volume is 1 meter, the natural gas bubbles cannot be transported out of the cavern when the flow rate of brine discharge is less than 60 m^3^/h. (4) If the distance between the lower end of the brine discharge pipe and the bottom of the gas storage is 1 meter, the flow rate of brine discharge should be small and slow.

**Fig 5:**
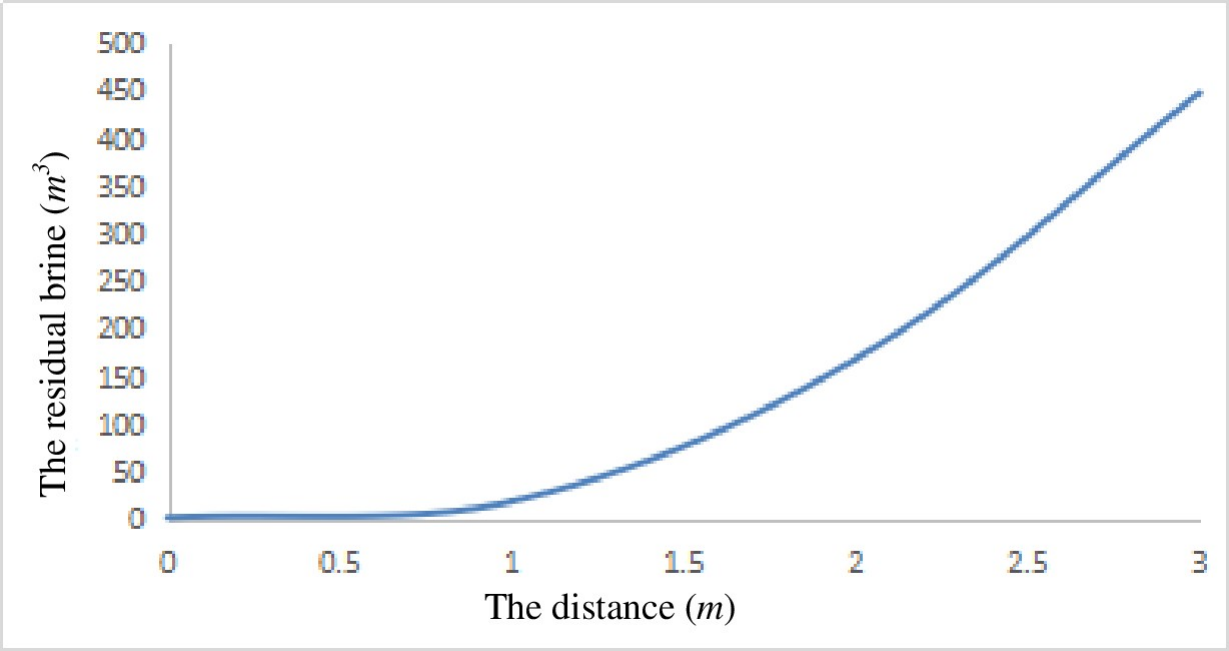
The relation curve between the residual brine and the distance from the lower end of the brine discharge pipe to the bottom of the gas storage volume.

The 3D rock salt geological model of the dissolved cavity was carried out by the geological modeling software, and the volume was calculated after the lower part of storage in the 1084–1088 m depth was converted to surface. The process of gas injection and brine discharge was simulated, and the results are shown in Fig 5. It can be seen that when the distance between the lower end of the brine discharge pipe and the surface of the brine is 2 meters, the following conclusions can be drawn: if the distance between the lower end of the brine discharge pipe and the bottom of the gas storage volume is 3 meters, the volume of the residual brine in the cavern is approximately 448 m^3^; if the distance between the lower end of the brine discharge pipe and the bottom of the gas storage volume is 2 meters, the volume of the residual brine in the cavern is approximately 168 m^3^; and if the distance between the lower end of the brine discharge pipe and the bottom of the gas storage volume is 1 meters, the volume of the residual brine in the cavern is approximately 18 m^3^, which can be considered the end of the brine discharge.

## 5 The effect of the residual brine on the gas injection and brine discharge

**Fig 6:**
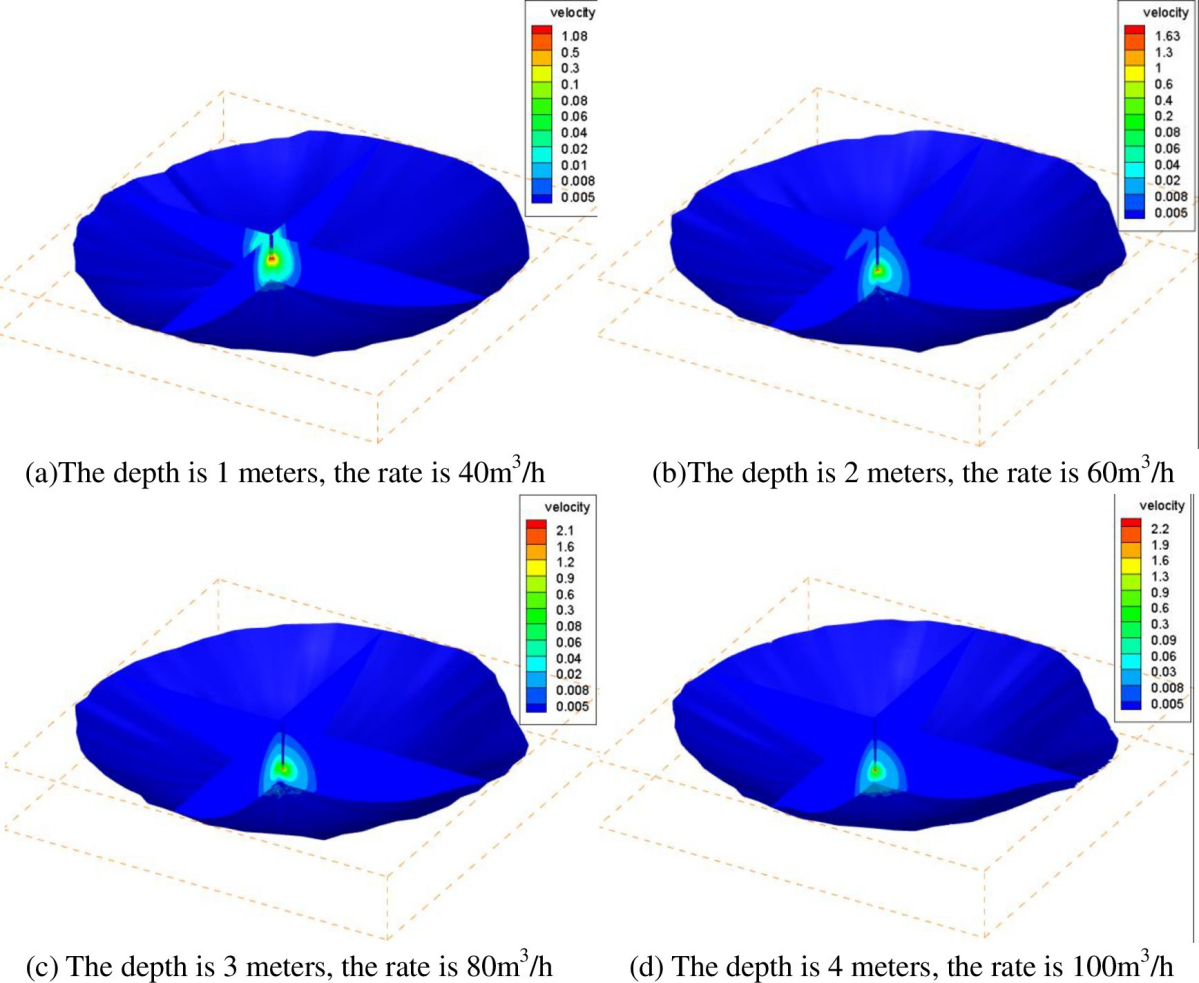
The velocity distribution of brine at the bottom of cavern under conditions of different depths of residual brine and different brine discharge flow rates. (a) The depth is 1 meter, and the rate is 40 m^3^/h. (b) The depth is 2 meters, and the rate is 60 m^3^/h. (c) The depth is 3 meters, and the rate is 80 m^3^/h. (d) The depth is 4 meters, and the rate is 100 m^3^/h.

In theory, the closer the bottom of the brine discharge pipe is, the less residual brine in the gas storage; however, the pipes cannot simply go down to the bottom of the cavern due to the safety and stability of the pipes. In this section, the four models of the lower part of the storage volume are all based on the actual working conditions when the distance from the lower end of brine discharge pipe to the bottom of the cavern is 1 meter. The movement trend of the fluid was calculated at the different flow rates when the depth of residual brine was 1 meters, 2 meters, 3 meters and 4 meters. According to the rule of brine flow rate optimization, Table 3 is the maximum flow rate on the residual brine surface for different depths of brine, and Fig 6 is the velocity profile of the corresponding brine flow.

**Table 3:** The maximum velocity on the surface of brine under different conditions.

Depth of residual brine (m)	Maximum velocity on the surface of brine (m/s)			
	Rate of fluid flow 100 m^3^/s	Rate of fluid flow 80 m^3^/s	Rate of fluid flow 60 m^3^/s	Rate of fluid flow 40 m^3^/s
1	0.0353	0.0277	0.0212	0.0144
2	0.0099	0.0079	0.0059	0.0039
3	0.0054	0.0043	0.0032	0.0022
4	0.0033	0.0027	0.0024	0.0013

Similar to previous research on the effects of pipe depth on gas injection and brine discharge, the natural gas bubble in the saturated brine moves upward with a velocity of *V*, and the saturated brine of the gas-liquid interface moves at a velocity of *Vw*, while the downward velocity component is *Vwy*. Because the velocity of the flow field gradually decreases from the lower end of the brine discharge pipe to the gas-liquid interface, only when *V* < *Vwy* can the bubbles be transported by the high-speed flow of brine. For example, to transport a natural gas bubble with diameter less than 0.1 mm, the maximum velocity of the gas-liquid interface must be less than 0.005 m/s.

In the actual situation of gas injection and brine discharge, the maximum rate of brine flow is controlled at 100 m^3^/h, and the average flow rate is approximately 80 m^3^/h due to the limitations of the ground equipment. Fig 6 shows the velocity distribution of brine at the bottom of the cavern under different conditions, and the depths of residual brine are 1 meter, 2 meters, 3 meters and 4 meters. Taking a bubble with a diameter of 0.1 mm as an example, the optimization of the working conditions can be carried out.

According to Table 3, taking the flow rate of 0.005 m/s as the standard, we can find the following: if the depth of brine that can be discharged into the gas storage is 4 meters, the maximum velocity of the brine interface is 0.0033 m/s when the rate of brine discharge is 100 m^3^/h, which means that it is safe to discharge the brine out of the gas storage; if the depth of brine that can be discharged into the gas storage is 3 meters, the maximum velocity of the brine interface is 0.0043 m/s when the rate of brine discharge is 80 m^3^/h, which means that it is safe to discharge the brine out of the gas storage; and if the depth of brine that can be discharged into the gas storage is 2 meters, the maximum velocity of the brine interface is 0.0059 m/s when the rate of brine discharge is 60 m^3^/h, which means that a small number of natural gas bubbles would be discharged with the brine out of the rock salt gas storage. It can be seen from Fig 6(A) that if there is only a 1 meter depth of brine in the cavern, the velocity near the pipe is larger than the rate limit when the rate of brine discharge is 40 m^3^/h; thus, a large number of bubbles are discharged with the brine, so the rate of the brine should be less than 20 m^3^/h to discharge the brine slowly out of the gas storage.

**Fig 7:**
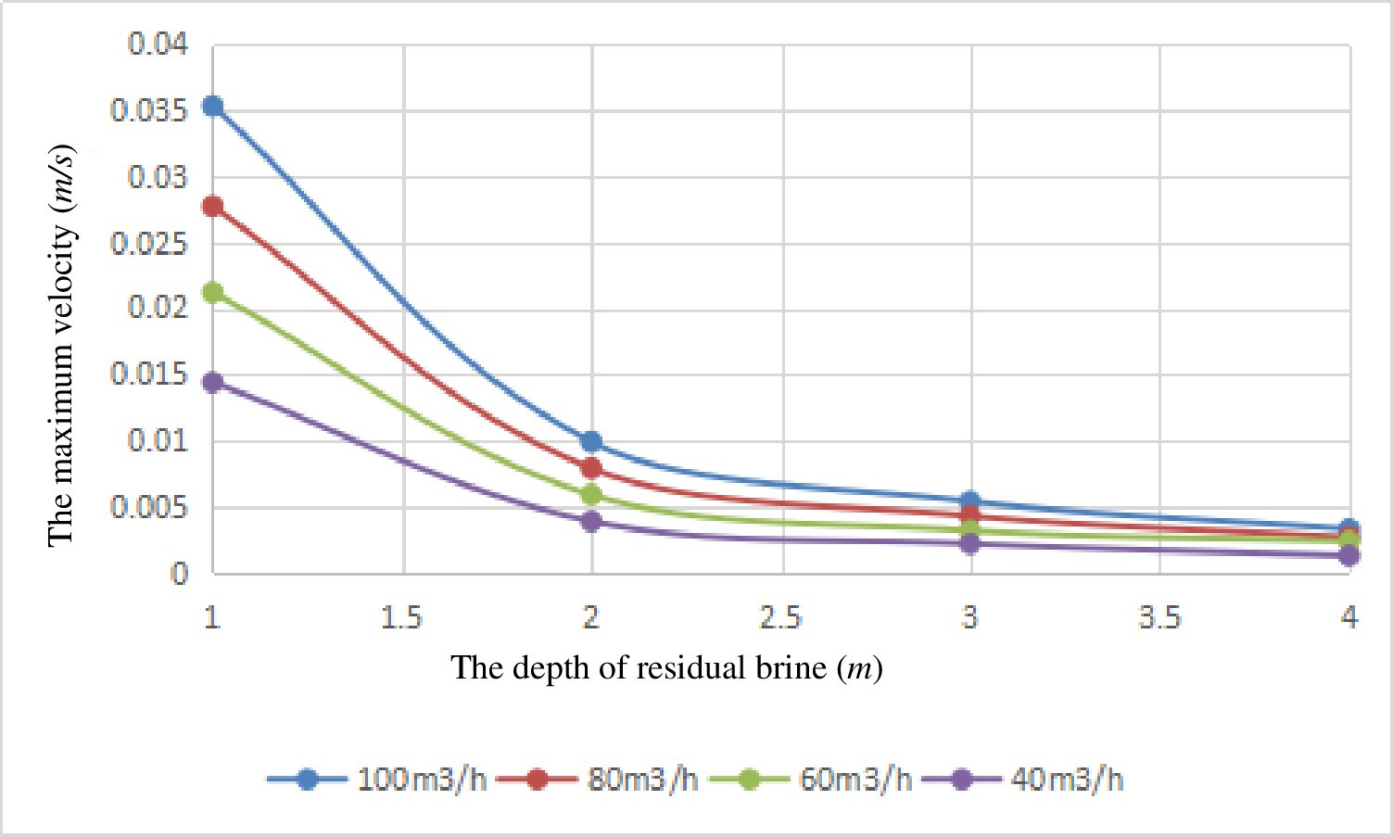
The relation curves between the maximum velocity on the surface of brine and the depth of the residual brine.

Fig 7 shows the relationship between the maximum velocity on the gas-liquid interface and the depth of the residual brine. It can be seen from the figure that the variation trends of the velocity curves under the four conditions are similar. Under each simulation condition, the velocity at the gas-liquid interface is the largest and the maximum velocity decreases linearly with the decrease in the flow rate. The maximum velocity of the gas-liquid interface increases gradually with the decrease of the brine surface, and the rate of change of the maximum velocity is the smallest when the depth of brine is 4 meters in the cavern. Compared with the velocity with the same amount of brine under different working conditions, as the depth of the residual brine is reduced, the maximum velocity rises increasingly more quickly.

## 6 Conclusion

When the rate of brine flow is held constant, the maximum velocity of the gas-liquid interface increases with the continuous deepening of the column, and its rate of change rate is increasing. For the same depth of brine pipe, the maximum velocity of the brine interface increases, and its increase is basically the same. When the discharge pipe is 1 meter from the bottom of the cavern, the effect of the residual brine content on the gas injection efficiency is discussed considering the factors of practical engineering. When the depths are 4 meters, 3 meters and 2 meters in the rock salt gas storage, the corresponding reasonable rates of brine discharge flow are 100 m^3^/h, 80 m^3^/h and 60 m^3^/h, respectively. When the depth is only 1 meter in the gas storage volume, it is reasonable to discharge the brine at a flow rate less than 20 m3/h. Due to the limitation of the quantity of gas storage at the present stage, the accuracy of velocity, distance and depth control can not be very high, the conclusions obtained in this study can only be verified in the existing gas storage cavity. But the conclusions of this research can still be the guideline in the engineering practice of natural gas storage.
